# Supplementary material for: Physical Inactivity Levels of European Adolescents in 2002, 2005, 2013, and 2017
Source: Int J Environ Res Public Health. 2023 Feb 20;20(4):3758. doi: 10.3390/ijerph20043758 (PMC9966786; doi:10.3390/ijerph20043758)
Supplement: Supplementary file 1 [file ijerph-20-03758-s001.zip › ijerph-2201946-supplementary.pdf]

Supplementary material

Supplementary file S1: STROBE Statement—checklist of items that should be included in reports of observational studies.

	Item No.	Recommendation	Page No.	Relevant text from manuscript
**Title and abstract**	1	(*a*) Indicate the study's design with a commonly used term in the title or the abstract	1	
		(*b*) Provide in the abstract an informative and balanced summary of what was done and what was found	1	
**Introduction**				
Background/rationale	2	Explain the scientific background and rationale for the investigation being reported	1,2	
Objectives	3	State specific objectives, including any prespecified hypotheses	2	The primary aim...
**Methods**				
Study design	4	Present key elements of study design early in the paper	3	2.1. Design
Setting	5	Describe the setting, locations, and relevant dates, including periods of recruitment, exposure, follow-up, and data collection	3	
Participants	6	(*a*) *Cohort study*—Give the eligibility criteria, and the sources and methods of selection of participants. Describe methods of follow-up<br>*Case-control study*—Give the eligibility criteria, and the sources and methods of case ascertainment and control selection. Give the rationale for the choice of cases and controls *Cross-sectional study*—Give the eligibility criteria, and the sources and methods of selection of participants	3	Data source
		(*b*) *Cohort study*—For matched studies, give matching criteria and number of exposed and unexposed<br>*Case-control study*—For matched studies, give matching criteria and the number of controls per case	3	Data source
Variables	7	Clearly define all outcomes, exposures, predictors, potential confounders, and effect modifiers.<br>Give diagnostic criteria, if applicable	3	Measures

Data sources/ measurement	8*	For each variable of interest, give sources of data and details of methods of assessment (measurement). Describe comparability of assessment methods if there is more than one group	3	Measures
Bias	9	Describe any efforts to address potential sources of bias	3	Measures
Study size	10	Explain how the study size was arrived at	3	Data source

Continued on next page

Quantitative variables	11	Explain how quantitative variables were handled in the analyses. If applicable, describe which groupings were chosen and why	3	
Statistical methods	12	(*a*) Describe all statistical methods, including those used to control for confounding	3	
		(*b*) Describe any methods used to examine subgroups and interactions	4	Statistical analysis
		(*c*) Explain how missing data were addressed	3	Measures
		(*d*) *Cohort study*—If applicable, explain how loss to follow-up was addressed *Case-control study*—If applicable, explain how matching of cases and controls was addressed *Cross-sectional study*—If applicable, describe analytical methods taking account of sampling strategy	3	Not applicable
		(*e*) Describe any sensitivity analyses	3	Not applicable
**Results**				
Participants	13*	(a) Report numbers of individuals at each stage of study—eg numbers potentially eligible, examined for eligibility, confirmed eligible, included in the study, completing follow-up, and analysed		Not applicable
		(b) Give reasons for non-participation at each stage		Not applicable
		(c) Consider use of a flow diagram		Not required
Descriptive data	14*	(a) Give characteristics of study participants (eg demographic, clinical, social) and information on exposures and potential confounders		Not applicable
		(b) Indicate number of participants with missing data for each variable of interest		Not applicable
		(c) *Cohort study*—Summarise follow-up time (eg, average and total amount)		Not applicable
Outcome data	15*	*Cohort study*—Report numbers of outcome events or summary measures over time	5	Table 1
		Case-control study—Report numbers in each exposure category, or summary measures of exposure		Not applicable
		Cross-sectional study—Report numbers of outcome events or summary measures	6	Table 1
Main results	16	(*a*) Give unadjusted estimates and, if applicable, confounder-adjusted estimates and their precision (eg, 95% confidence interval). Make clear which confounders were adjusted for and why they were included	6	Table 1
		(*b*) Report category boundaries when continuous variables were categorized	6	Table 1
		(*c*) If relevant, consider translating estimates of relative risk into absolute risk for a meaningful time period		Not applicable

Continued on next page

Other analyses	17	Report other analyses done—eg analyses of subgroups and interactions, and sensitivity analyses	3-6	Statistical analysis, Results, Table 1
**Discussion**				
Key results	18	Summarise key results with reference to study objectives	7	This study revealed...
Limitations	19	Discuss limitations of the study, taking into account sources of potential bias or imprecision. Discuss both direction and magnitude of any potential bias	9	
Interpretation	20	Give a cautious overall interpretation of results considering objectives, limitations, multiplicity of analyses, results from similar studies, and other relevant evidence	9	Results
Generalisability	21	Discuss the generalisability (external validity) of the study results	8	The present study evidence
**Other information**				
Funding	22	Give the source of funding and the role of the funders for the present study and, if applicable, for the original study on which the present article is based	9	Funding

*Give information separately for cases and controls in case-control studies and, if applicable, for exposed and unexposed groups in cohort and cross-sectional studies.

Note: An Explanation and Elaboration article discusses each checklist item and gives methodological background and published examples of transparent reporting. The STROBE checklist is best used in conjunction with this article (freely available on the Web sites of PLoS Medicine at http://www.plosmedicine.org/, Annals of Internal Medicine at http://www.annals.org/, and Epidemiology at http://www.epidem.com/). Information on the STROBE Initiative is available at www.strobe-statement.org.

Supplementary file S2. Data from all studied EU countries.

	**2002**			**2005**			**2013**			**2017**		
	**Sample**	**PIA (%)**	**95% CI**	**Sample**	**PIA (%)**	**95% CI**	**Sample**	**PIA (%)**	**95% CI**	**Sample**	**PIA (%)**	**95% CI**
Austria	33	81.8	67.9-93.9	21	81.0	63.0-95.7	13	76.9	50.0-100.0	8	75.0	40.0-100.0
Belgium	36	69.4	53.3-83.7	36	66.7	51.4-81.1	30	76.7	60.0-90.3	16	87.5	66.7-100.0
Bulgaria	Not UE member in 2002			46	60.9	45.5-74.5	14	92.9	76.5-100.0	23	95.7	86.2-100.0
Croatia	Not UE member in 2002			32	68.8	52.9-85.3	9	77.8	42.9-100.0	12	91.7	72.7-100.0
Cyprus Republic	Not UE member in 2002			29	79.3	64.0-93.3	15	86.7	66.7-100.0	14	85.7	66.7-100.0
Czech Republic	Not UE member in 2002			33	45.5	30.0-63.3	20	85.0	66.7-100.0	10	90.0	66.7-100.0
Denmark	10	80.0	50.0-100.0	20	60.0	37.5-81.3	18	66.7	41.7-88.2	15	73.3	50.0-94.4
Estonia	Not UE member in 2002			44	56.8	41.0-70.8	19	63.2	41.2-83.3	19	68.4	45.0-88.9
Finland	42	71.4	57.5-85.3	35	74.3	58.1-88.6	16	81.3	58.9-100.0	16	75.0	50.0-94.7

France	21	71.4	50-91.7	26	84.6	68.2-96.4	19	68.4	46.2-87.5	23	87.0	72.7-100.0
Germany	41	53.7	38.2-68.3	43	55.8	40.0-70.7	32	75.0	57.9-90.0	14	78.6	52.9-100.0
Great Britain	20	65.0	42.9-8.50	27	66.7	46.4-85.0	25	96.0	87.0-100.0	16	75.0	52.6-93.8
Greece	28	82.1	66.7-95.0	21	52.4	30.8-75.0	27	66.7	47.6-84.8	26	88.5	74.1-100.0
Hungary	Not UE member in 2002			25	56.0	37.5-78.1	15	80.0	57.9-100.0	3	100	0.0-100.0
Italy	21	76.2	56.3-94.1	30	80.0	65.4-93.5	6	83.0	50.0-100.0	2	100	0.0-100.0
Ireland	49	73.5	61.0-85.4	29	86.2	71.4-96.9	28	82.1	66.7-96.1	17	82.4	61.5-100.0
Latvia	Not UE member in 2002			60	71.7	60.6-82.7	31	71.0	53.7-86.8	29	69.0	52.0-85.2
Lithuania	Not UE member in 2002			52	65.4	52.2-78.3	43	74.4	60.0-86.5	15	73.3	50.0-95.4
Luxembourg	19	68.4	47.1-89.5	29	51.7	33.3-70.4	19	78.9	60.0-95.0	19	57.9	33.4-78.9
Malta	Not UE member in 2002			17	94.1	81.3-100.0	3	66.7	0.0-100.0	6	83.3	43.0-100.0
Poland	Not UE member in 2002			50	58.0	45.2-72.1	12	91.7	71.5-100.0	16	56.3	31.3-81.8
Portugal	45	66.7	53.7-80.4	31	64.5	48.0-81.5	14	71.4	46.8-93.8	19	84.2	66.7-100.0
Romania	Not UE member in 2002			42	64.3	48.8-79.2	21	85.7	69.6-100.0	26	84.6	69.6-96.7
Slovakia	Not UE member in 2002			13	61.5	33.3-85.7	19	89.5	73.7-100.0	3	66.7	0.0-100.0
Slovenia	Not UE member in 2002			45	73.3	60.0-85.7	17	64.7	42.9-85.7	26	80.8	65.0-95.5
Spain	49	73.5	61.1-85.2	35	82.9	68.4-94.6	21	81.0	63.6-95.8	19	68.4	46.7-90.0
Sweden	12	91.7	73.3-100.0	33	97.0	90.0-100.0	20	65.0	42.9-85.2	2	100	0.0-100.0
The Netherlands	36	66.7	51.4-82.9	35	42.9	26.7-60.0	35	68.6	51.3-83.8	38	52.6	36.0-68.4

CI: Confidence intervals.
